# A New Leaf Bacterial Disease of Foxtail Millet Caused by *Pantoea ananatis* in China

**DOI:** 10.3390/plants15192986

**Published:** 2026-09-30

**Authors:** Ning Han, Guo-Qing Yu, Xi-Wang Ke, Li-Wen Zhuang, Hong-Xi Sun, Jin-Hao Lu, Liang Ren, Yu-Hu Zuo

**Affiliations:** 1 College of Agriculture, Heilongjiang Bayi Agricultural University, Daqing 163319, China; hanning202106@163.com (N.H.);; 2 Liaoning Institute Sandy Land Control and Utilization, Fuxin 123000, China

**Keywords:** foxtail millet, *Pantoea ananatis*, plant protection, identification, genome, host colonization

## Abstract

Long-term continuous monocropping of foxtail millet (*Setaria italica*) in China has increased crop vulnerability to emerging diseases and threatens yield stability and grain quality. This practice may also influence inoculum carryover and host–pathogen interactions, making foxtail millet health a central issue for sustainable production. This study reports the first occurrence in China of a previously undescribed bacterial leaf disease of foxtail millet. Early symptoms are characterized by water-soaked lesions at leaf tips that spread basipetally along the veins and gradually desiccate as the tissue dehydrates. Koch’s postulates were fulfilled to isolate and identify the causal agent. Multilocus sequence analysis of three concatenated housekeeping genes (*gyrB*, *infB*, and *rpoB*) classified the pathogen as *Pantoea ananatis*. The isolate was further characterized for metabolic profile; its colonization pattern and spatial distribution within foxtail millet tissues were elucidated, revealing features of host–pathogen interaction. A high-quality de novo whole-genome assembly was generated, and candidate virulence determinants were identified by integrated genome annotation and cell wall hydrolase activity assays. This genomic resource is intended as a supporting tool for plant protection research, including disease surveillance, resistance breeding, and management. Collectively, this study represents the first report of *P. ananatis* causing bacterial leaf disease on foxtail millet in China. It clarifies the colonization and distribution of the pathogen in host tissues, reveales reveals its interaction characteristics with foxtail millet, and provided genomic resources to support research on foxtail millet health protection and plant protection under continuous cropping conditions.

## 1. Introduction

Foxtail millet (*Setaria italica*), domesticated in China, ranks among China’s oldest cultivated cereals, with a history spanning approximately 8000 to 10,000 years [1]. China dominates global foxtail millet production and consumption. Production is concentrated in the arid and semi-arid regions of North, Northwest, and Northeast China, most notably Hebei, Shanxi, Inner Mongolia, Shaanxi, Liaoning, and Gansu provinces [2]. In recent years, China’s annual sown area has ranged from 0.7 to 0.9 million hectares, with total production between 1.5 and 2.0 million tonnes, accounting for approximately 70% and 80% of global area and output, respectively [3]. The crop combines drought tolerance, high water use efficiency, and a short growth cycle, making it uniquely suited to rainfed and marginal croplands with limited irrigation [2,4]. Its grain is rich in protein, vitamins, minerals, and phenolic bioactive compounds and is regarded as a nutritionally valuable coarse cereal; the straw also provides high-quality forage, enabling a dual grain and fodder system [3,4]. These traits collectively position foxtail millet as a strategic crop for food security, cropping system diversification, water-saving agriculture, and rural revitalization [2].

However, under long-term continuous monocropping, foxtail millet health has become increasingly vulnerable to disease [5,6]. Blast, downy mildew, rust, and smut, caused by diverse fungal and oomycete pathogens, are widespread in production fields and can substantially reduce yields in severe years [4]. Continuous monocropping may exacerbate pathogen carryover and selection pressure, while host–pathogen interactions in this system remain insufficiently understood [5,6]. A detailed understanding of the identity, distribution, and pathogenic variability of the dominant pathogens in China’s major production regions is therefore critical to resistance breeding and integrated disease control.

Bacterial pathogens are persistent threats to crop health and productivity in global agricultural ecosystems. *Pantoea ananatis* has become an especially threatening pathogen because it infects a remarkably broad spectrum of crop species that hold substantial economic value [7]. Initially identified in 1928 on pineapple in the Philippines [8], *P. ananatis* has since been reported to infect numerous monocotyledonous and dicotyledonous plants, leading to sporadic outbreaks and significant economic losses [7].The increasing incidence of diseases associated with *P. ananatis*, coupled with its expanding host spectrum and geographical distribution, has firmly established it as an emerging plant pathogen [7,9]. The bacterium possesses a remarkably wide host range, having been documented on rice [10,11,12,13,14], maize [15,16,17,18,19], wheat [20,21], and many other economically important crops [22,23,24,25]. These records highlight the ability of *P. ananatis* to adapt to diverse host environments and to interact with different plant tissues.

Genomic studies have shown that *P. ananatis* lacks many canonical virulence determinants found in well-studied plant pathogens, including *Pseudomonas*, *Xanthomonas*, and *Ralstonia*, as well as determinants typical of enteric bacterial plant pathogens, including type II and Hrp type III secretion systems and phytotoxins [7,26]. Nevertheless, many *P. ananatis* strains also possess genes corresponding to an array of additional virulence-related candidates, among them those directing the assembly of flagella and pili, adhesins independent of pili, a carbohydrate polymer similar to amylovoran, and enzymes predicted to compromise the plant cell wall [26,27,28].

Recent genetic and mutagenesis studies have advanced understanding of *P. ananatis* pathogenesis. For example, strains can harbor up to three type VI secretion system (T6SS) gene clusters, which likely contribute to virulence in both animal and plant hosts [29,30]. The first and second T6SS loci are common in *P. ananatis* and are thought to play roles in antibacterial activity, fitness, and niche adaptation [30]. The second, incomplete T6SS locus probably evolved from the first via duplication, while the third was obtained through HGT [29]. Shyntum et al. [31] demonstrated that the first T6SS cluster is entirely functional in plant hosts such as onion seedlings and in intra- and interspecific bacterial competition.

Despite its recognition as a significant plant pathogen, the specific mechanisms by which *P. ananatis* causes disease on foxtail millet remain poorly understood. Unraveling the genetic basis of its pathogenicity in millet is critical. This forms the foundation for developing effective disease control strategies. Genome sequencing and comparative analysis provide powerful approaches for uncovering genetic determinants of pathogenicity and for dissecting pathogen–host molecular interactions [32,33,34].

This study provides the first formal confirmation via Koch’s postulates of the etiological agent responsible for a foliar disease of foxtail millet in Liaoning Province, China. Although multilocus sequence analysis of housekeeping genes identified the pathogen as *P. ananatis*, molecular diagnostics alone cannot resolve its precise infection behavior within host tissues or the underlying genetic basis of its virulence. To address this gap, the biochemical properties of the pathogen were characterized, and its colonization and spatial distribution within foxtail millet were mapped, revealing key phenotypic features of the infection cycle. To further dissect the genetic determinants of pathogenicity, Oxford Nanopore long-read sequencing was used to assemble a complete, high-quality genome of this isolate. A suite of virulence-associated genes involved in infection structure formation, toxin biosynthesis, and secretion systems was systematically annotated. This genome serves as a supporting resource for plant protection research. These findings establish a theoretical basis for optimizing field management and chemical control strategies for this disease in foxtail millet, lending support to plant protection practice.

## 2. Results

### 2.1. Occurrence, Symptomatology and Pathogen Identification

**Figure 1:**
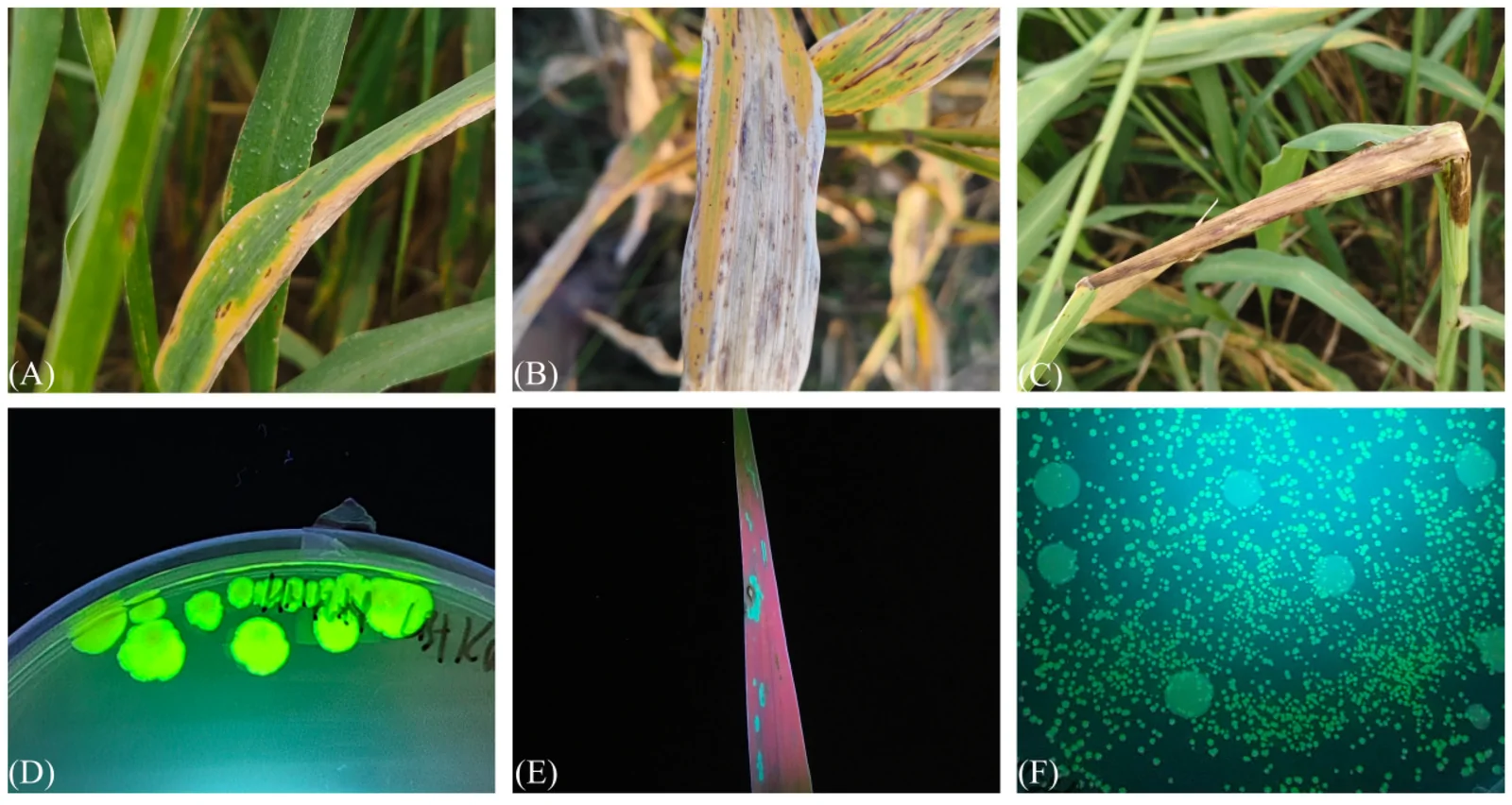
Symptoms and etiological characterization of a novel disease affecting *Setaria italica* (foxtail millet). (**A**) Early-stage foliar symptoms on infected plants. (**B**) Advanced foliar manifestations during disease progression. (**C**) Symptomatology observed on culms (stems) of diseased plants. (**D**) The strain of FXFM-GFP. (**E**) The symptomatic response following controlled inoculation under greenhouse conditions, shown on leaf panels. (**F**) The re-isolate.

In 2022, a previously unreported leaf disease of foxtail millet was detected in field plots located in Fuxin City, Liaoning Province, China. Early-stage symptoms featured water-soaked lesions emerging at leaf tips that progressively extended basipetally along the leaf margins. Subsequently, the affected leaf edges desiccated and developed a pale greyish discoloration (Figure 1A). During the late disease phase, entire leaves exhibited curling and complete necrosis, accompanied by stem rot in a subset of infected plants (Figure 1B,C). These strongly implicated a bacterial etiology. Conventional tissue-isolation procedures were employed to recover putative pathogens from diseased leaf tissues, and purified colonies were obtained via serial dilution and axenic culture. A bacterial suspension of the GFP-tagged FXFM strain was applied to the foxtail millet cultivar ‘Jinmiao K1’ via soil drench inoculation. By 13 dpi post inoculation, all inoculated plants developed visible symptoms, yielding a disease incidence of 100%. Re-isolation from symptomatic leaves yielded the GFP-tagged strain, whose colony morphology was identical to that of the inoculum (Figure 1D–F). Together, these inoculation and re-isolation results fulfilled Koch’s postulates, identifying the isolate as the causal agent of this novel bacterial disease. The strain was designated FXFM.

### 2.2. Molecular Identification of the Pathogen

**Figure 2:**
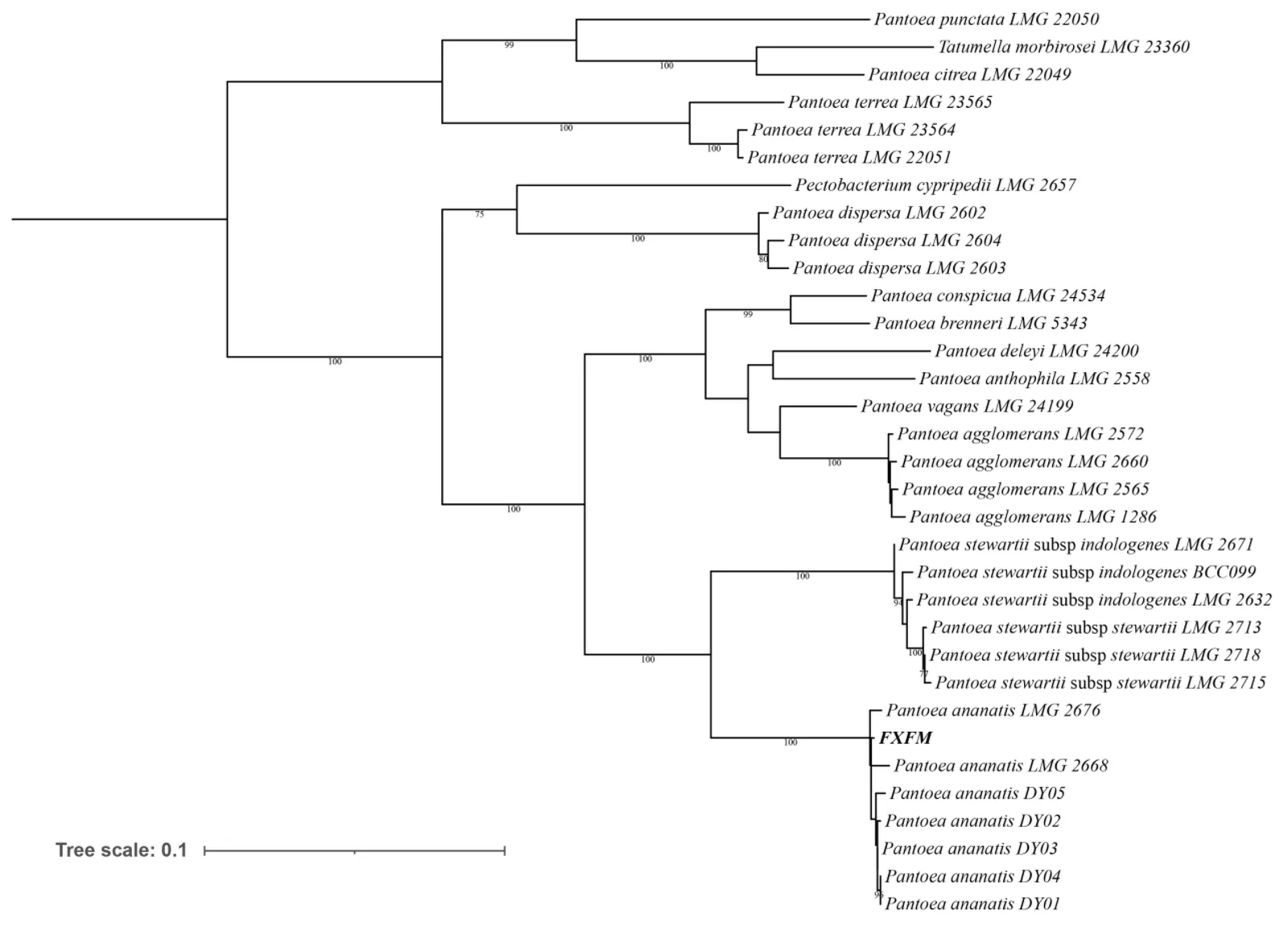
Phylogenetic tree inferred from the concatenated nucleotide sequences of the *gyrB*, *infB* and *rpoB* genes. The evolutionary relationships were inferred using a maximum-likelihood approach. The values at the nodes indicate bootstrap support from 1000 replications in the maximum likelihood analysis performed with IQ-TREE.

To resolve the phylogenetic placement of isolate FXFM within the genus *Pantoea*, three housekeeping genes were targeted for analysis. The *gyrB*, *infB*, and *rpoB* loci were amplified via PCR and subsequently sequenced. A concatenated phylogenetic tree was constructed using the combined sequences of these three housekeeping genes across multiple *Pantoea* species. Phylogenetic reconstruction demonstrated that isolate FXFM clustered robustly within the *P. ananatis* clade, indicating its closest genetic affinity to this species (Figure 2).

### 2.3. Biochemical Characterization of the Causal Agent

**Figure 3:**
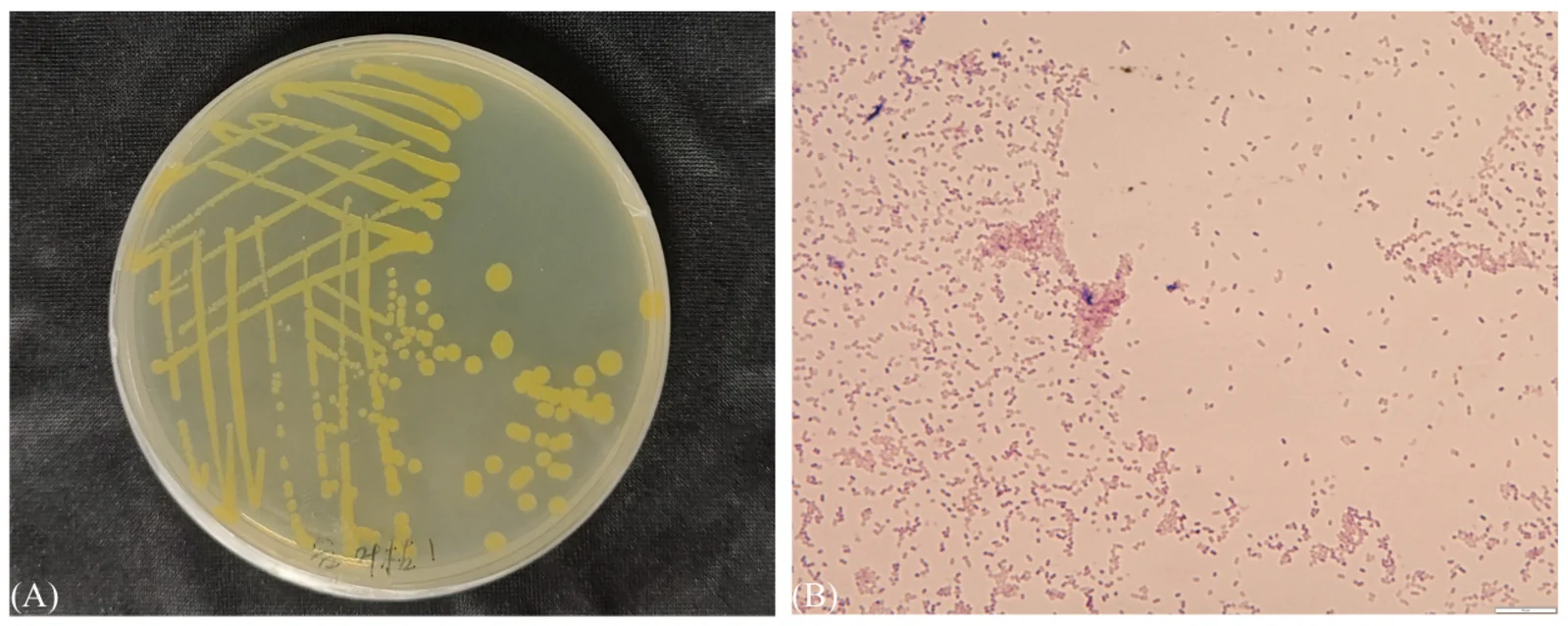
Morphology of the pathogenic bacterium. (**A**) Colony morphology of the pathogen following cultivation on LB medium for 2 days. (**B**) Gram-staining profile of the pathogenic isolate (bar = 10 μm).

The taxonomic assignment of the pathogen was preliminarily established by molecular markers. The isolate formed circular, smooth, yellow-pigmented colonies. Microscopic examination revealed a rod-shaped morphology, and Gram staining classified the bacterium as Gram-negative (Figure 3). Notably, the isolate exhibited an oxidase-negative, arginine dihydrolase-positive phenotype, together with the capacity to utilize maltose, mannitol, and xylose, whereas no metabolic activity was detected with glucose (Table 1). This biochemical profile supports the taxonomic identification and offers insight into the nutritional preferences and survival strategies of this isolate.

### 2.4. Colonization and Distribution of P. ananatis in Foxtail Millet Plants

**Figure 4:**
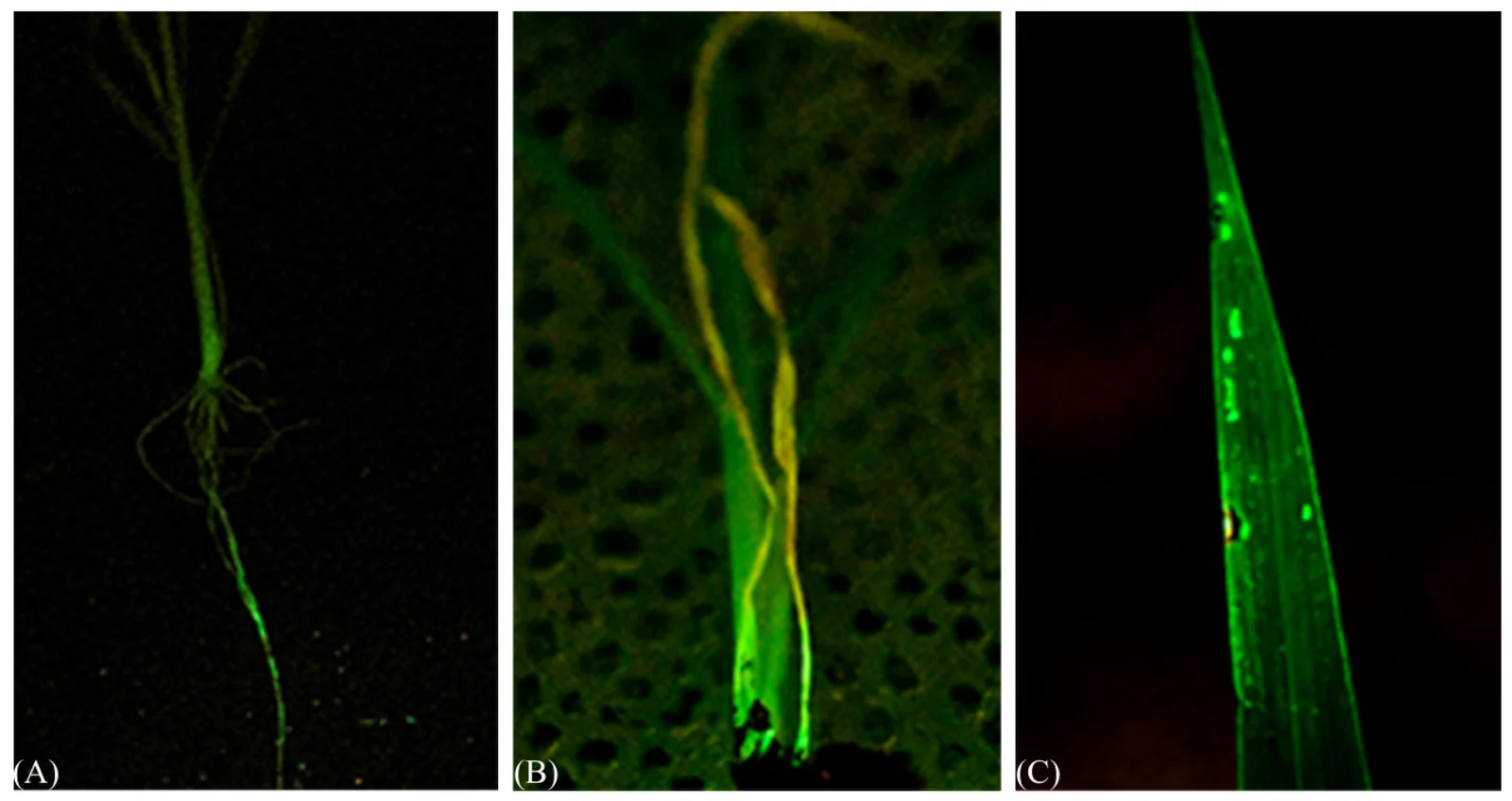
Colonization and distribution of the pathogen in foxtail millet. (**A**–**C**): Visualization of pathogen colonization in the roots, stems and leaves of foxtail millet, respectively.

To elucidate the infection route and colonization dynamics of *P. ananatis* within its host, GFP-tagged bacterial cells were introduced into foxtail millet seedlings via soil drench inoculation. Fluorescence observation was subsequently performed at 13 days post-inoculation. Intense GFP fluorescence was detected not only at the inoculation site (roots) but also prominently in the above-ground tissues of infected seedlings, including the leaf sheaths and vascular bundles of leaves (Figure 4). These observations demonstrate that *P. ananatis* is capable of upward translocation from the root infection court to aerial tissues via internal host structures, typified by the vascular system, and establishes successful colonization therein. The widespread distribution of the pathogen across distinct host tissues unequivocally confirms that *P. ananatis*-induced foxtail millet disease represents a canonical systemic infection.

### 2.5. Genome Sequencing, Assembly and Functional Annotation

#### 2.5.1. Complete Genome Assembly and Analysis of *P. ananatis*

**Figure 5:**
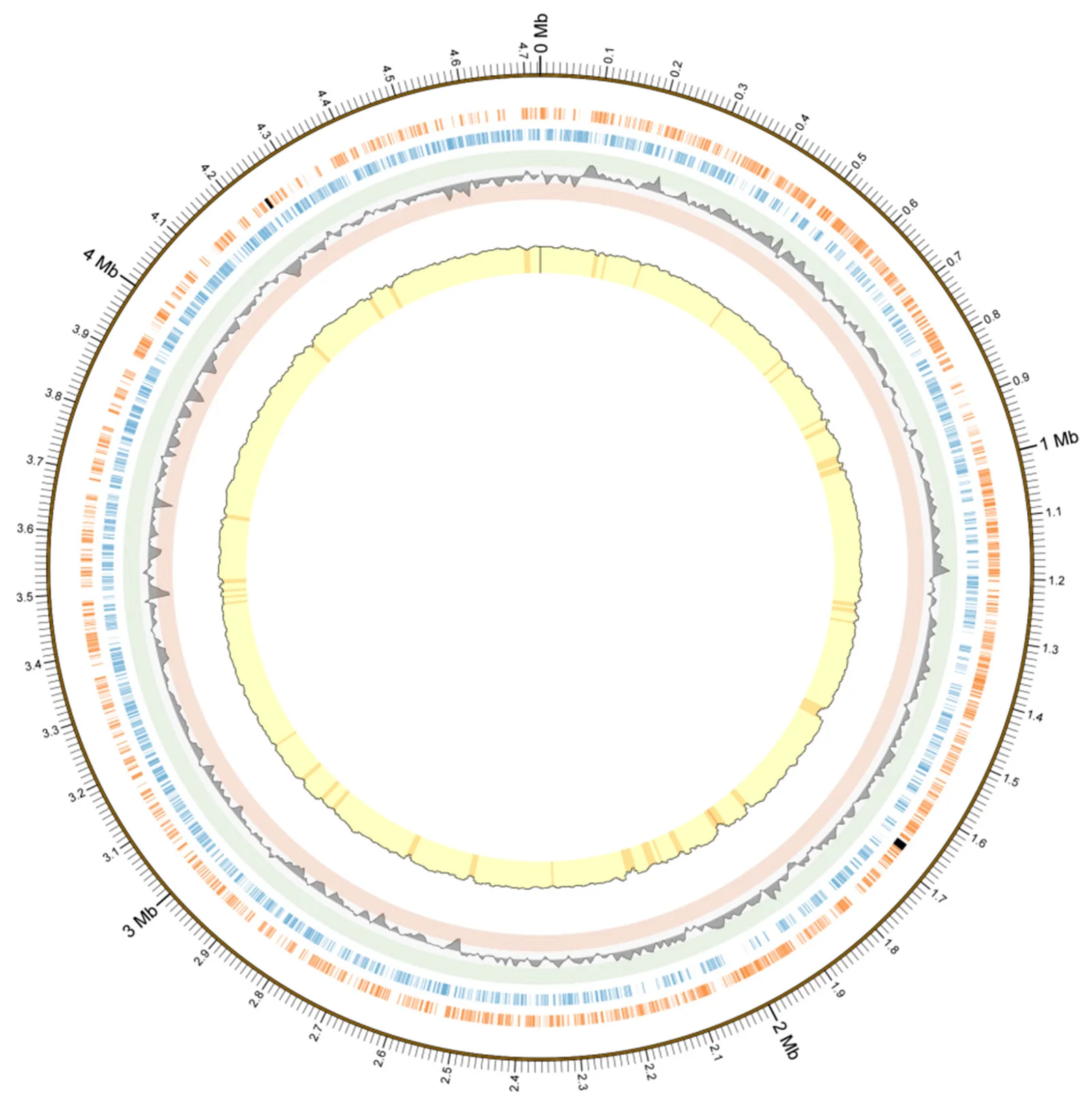
Circular genome map. From the outermost to the innermost circle: the first circle represents the genomic sequence information. The second circle depicts the coding sequences (CDS) and non-coding RNA regions (rRNA and tRNA) annotated against the reference genome, with the outer and inner layers indicating the positive and negative strands, respectively. The third circle shows the GC skew profile, calculated using a sliding window of 2000 bp; the dashed line indicates the baseline where GC skew equals zero. The fourth circle presents the GC content profile, also computed with a 2000 bp sliding window; the dashed line denotes the average GC content of the reference genome.

The FXFM isolate was subjected to whole-genome sequencing. After removal of adaptors, low-quality reads and short fragments (<2000 bp), 11,677,250 high-quality clean reads were generated. These reads yielded 1,743,553,882 bp (approximately 1.74 Gb) of clean bases, corresponding to an approximate 369× sequencing coverage relative to the final assembled genome size. De novo assembly produced a gap-free complete chromosomal genome along with one plasmid. BUSCO assessment using the bacteria odb10 database confirmed the high completeness of this assembly, with 100% complete BUSCOs (99.2% single-copy and 0.8% duplicated) and no fragmented or missing genes among the 124 conserved single-copy orthologs searched. Plasmidome sequencing revealed that the isolate harbours a circular plasmid of 304,093 bp with an overall GC content of 52.0%. The sequencing depth across this region reached only 0.72×. This observation implies either a low copy number for the replicon in the host strain or insufficient enrichment of plasmid-derived sequences during sequencing (Table 2). The complete genome size was determined to be 4,724,278 bp, with an assembled scaffold N50 length of 4,724,278 bp. Based on the genome assembly, a total of 4423 genes were predicted, comprising 4340 protein-coding genes and 83 tRNA genes. Genes averaged 926 bp in length, and the GC content within gene regions was 54%. Gene density was calculated as 963 genes per megabase, and the coding sequences accounted for 87% of the genome, leaving an intergenic region length of 602,868 bp (Figure 5).

#### 2.5.2. Gene Annotation

**Figure 6:**
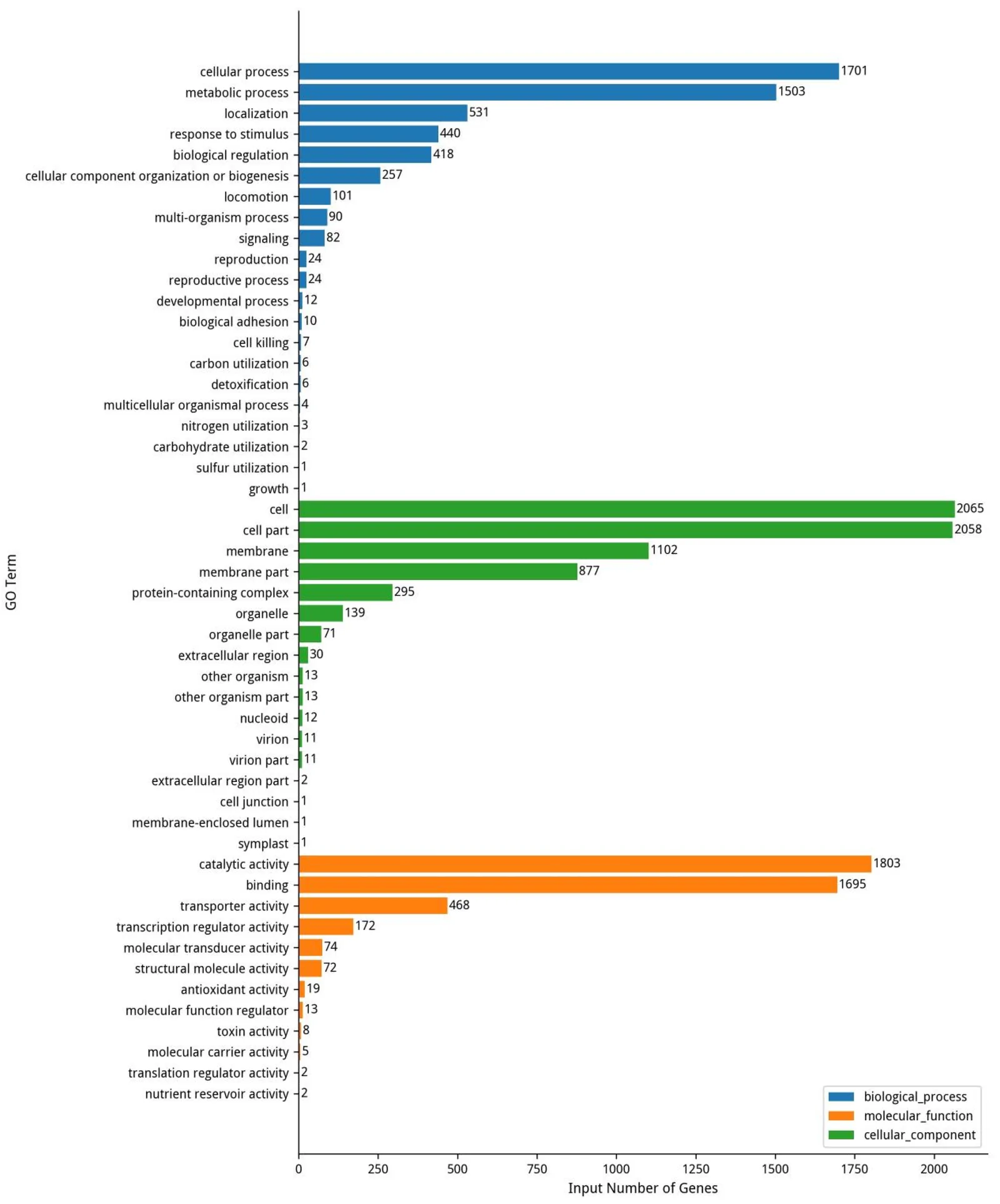
GO annotation classification statistics. Gene Ontology functional groups are displayed along the x-axis; the left y-axis provides the percentage of genes, while the right y-axis gives the corresponding gene count.

Based on Gene Ontology (GO) annotation analysis, a total of 3145 genes of the pathogenic bacterium were functionally annotated. The biological process category displayed enrichment mainly in cellular process (1701), metabolic process (1503), localization (531), response to stimulus (440), and biological regulation (418). Among these enriched terms, genes linked to cellular and metabolic processes accounted for the highest proportion. Genes related to localization, biological regulation, and response to stimulus provided genetic support for its environmental adaptation and physiological regulation. In terms of molecular function, catalytic activity was the dominant term, with 1803 genes accounting for 57.33% of the total. This finding strongly confirms the organism’s robust metabolic capacity and its ability to degrade cell wall components. Genes related to binding (1695) and transporter activity (468, including ABC transporters) were also abundantly represented. These genes collectively provide a solid foundation for signal transduction, substance uptake, and secretion. Cellular component analysis showed that a great many genes were localized to the cell component (2065), cell part (2058), and membrane (1102), indicating that the membrane system plays a crucial role in environmental sensing, nutrient exchange, and virulence factor secretion (Figure 6). Collectively, the genome of this bacterium encodes abundant catalytic enzymes and membrane proteins, as well as genes related to pathogenicity, chemotaxis, motility, and iron acquisition, which together constitute the molecular basis for its environmental adaptability and potential pathogenicity.

**Figure 7:**
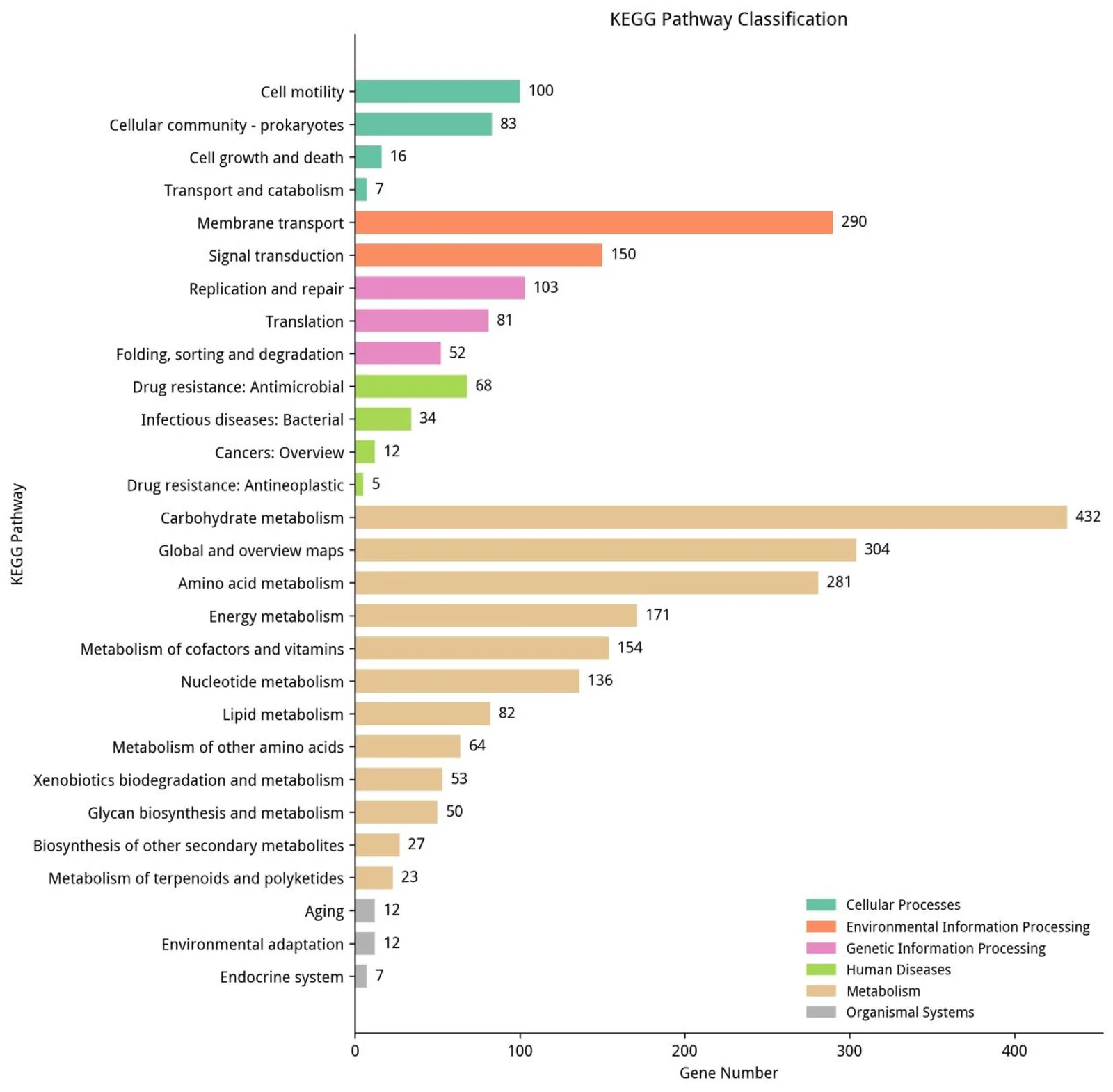
KEGG annotation classification statistics. KEGG categories are listed on the y-axis, while the x-axis indicates the number of genes.

Based on annotation in the KEGG database, a total of 2809 genes of the pathogenic bacterium were mapped to 28 pathways. Metabolic-related pathways were the most enriched, with 1777 genes associated with these pathways. Among these enriched metabolic pathways, the carbohydrate metabolism pathway included 432 genes, which encompass central metabolic routes including glycolysis and the pentose-phosphate shunt. In total, 281 genes were linked to the amino acid metabolism pathway, encompassing the majority of amino acid synthesis and degradation pathways. Additionally, 171 genes were involved in energy metabolism, including key pathways such as oxidative phosphorylation as well as nitrogen and sulfur metabolism. In terms of environmental information processing, the membrane transport pathway comprised 290 genes. Detailed KEGG annotation revealed that the ABC transporters pathway (218 genes) was the most dominant, while the intact two-component system pathway (145 genes) underpinned environmental adaptation and pathogenic regulation. In cellular processes, the identification of pathways related to cell motility (100 genes) indicated that the bacterium possesses motility and chemotaxis capabilities (Figure 7). In summary, KEGG analysis revealed that the bacterium has a complete central metabolic network and is enriched in pathways related to nutrient uptake (ABC transporters), signal transduction (two-component systems), motility (cell motility), and pathogenicity, providing molecular evidence for its environmental adaptation and potential pathogenic mechanisms.

**Figure 8:**
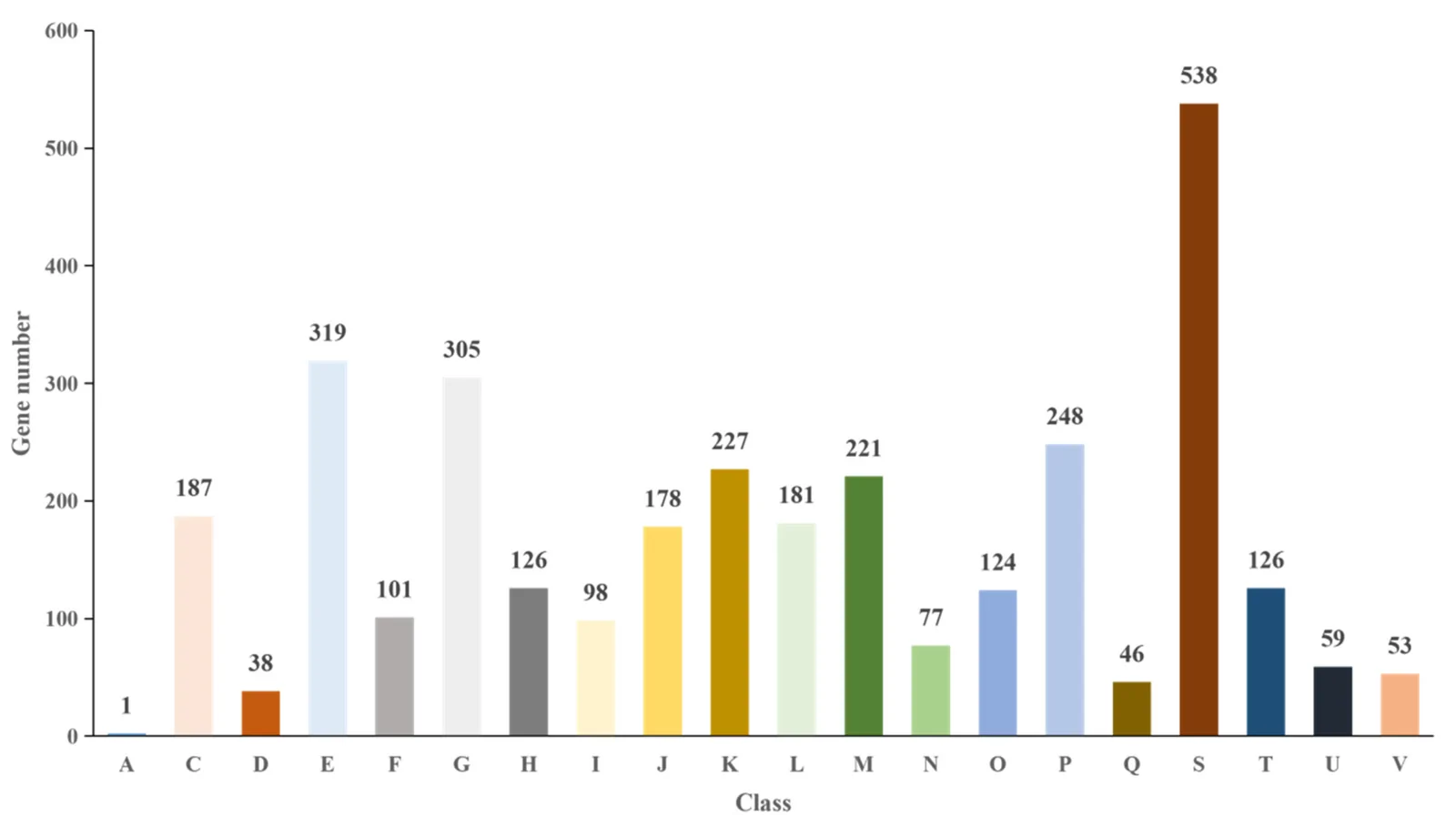
EggNOG/COG annotation classification statistics. The horizontal axis represents eggNOG/COG functional categories, and the vertical axis represents the gene count. A: RNA processing and modification, C: Energy production and conversion, D: Cell cycle control, cell division, chromosome partitioning, E: Amino acid transport and metabolism, F: Nucleotide transport and metabolism, G: Carbohydrate transport and metabolism, H: Coenzyme transport and metabolism, I: Lipid transport and metabolism, J: Translation, ribosomal structure and biogenesis, K: Transcription, L: Replication, recombination and repair, M: Cell wall/membrane/envelope biogenesis, N: Cell motility, O: Posttranslational modification, protein turnover, chaperones, P: Inorganic ion transport and metabolism, Q: Secondary metabolites biosynthesis, transport and catabolism, S: Function unknown, T: Signal transduction mechanisms, U: Intracellular trafficking, secretion, and vesicular transport, V: Defense mechanisms.

Based on annotation in the eggNOG database, 4071 coding sequences (CDSs) were identified in the whole genome of the pathogenic bacterium, among which 3148 CDSs obtained clear functional information, with an annotation success rate of 77.3%. The remaining 923 unannotated CDSs accounted for 22.7% of the total. These unannotated CDSs were mainly hypothetical proteins, with some being strain-specific or novel functional genes that have not yet been included in the database. The annotated genes covered 21 major COG functional categories. Among these categories, the top 5 by proportion were Function unknown (17.1%), Amino acid transport and metabolism (10.1%), Carbohydrate transport and metabolism (9.7%), Inorganic ion transport and metabolism (7.9%), and Transcription (7.2%). Among the functional categories closely related to pathogenicity, genes involved in intracellular trafficking, secretion, and vesicular transport accounted for 1.9%, those related to defense mechanisms accounted for 1.7%, and those associated with secondary metabolite biosynthesis accounted for 1.5%, with virulence regulators and drug resistance-related genes all annotated. Additionally, genes related to energy and substance metabolism (These functional categories comprised energy biogenesis, together with the trafficking and metabolic conversion of amino acids, nucleotides, carbohydrates, coenzymes, lipids, and mineral ions) totaled 1384, accounting for 44.0%, providing sufficient material and energy support for the pathogen during host infection (Figure 8).

#### 2.5.3. Prediction of Secretory Proteins

**Figure 9:**
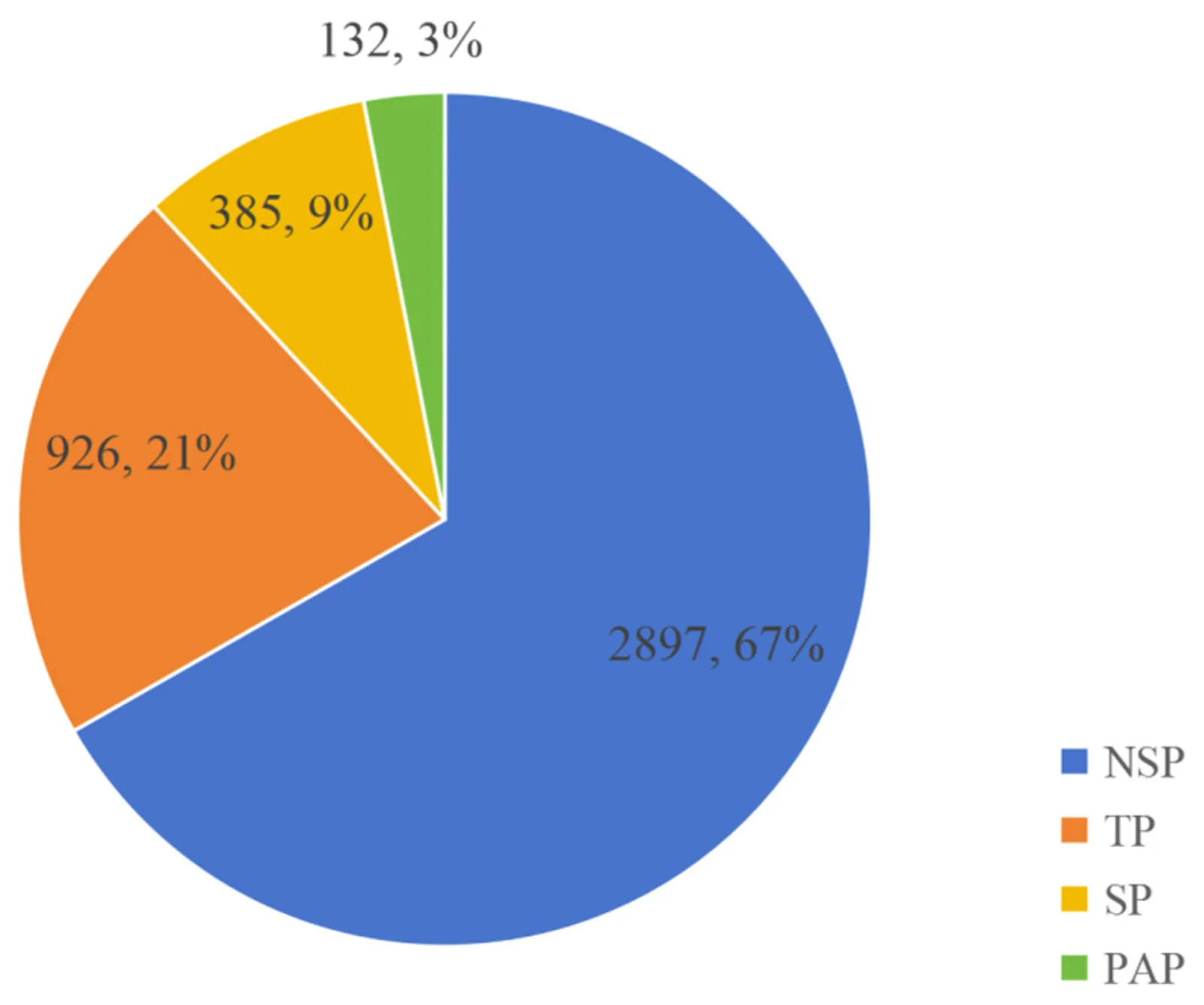
Secretory proteins annotation. NSP: Soluble non-secretory proteins, TP: Transmembrane proteins, SP: Secretory proteins, PAP: Signal peptide-anchored membrane proteins.

To enhance virulence, phytopathogens secrete diverse effector repertoires. Many apoplastic effectors produced by plant-pathogenic fungi play critical roles in suppressing plant immunity, promoting pathogenicity and inducing host cell death [35,36,37]. In this study, systematic secretome profiling of proteins encoded by the pathogen genome was performed through the integrated implementation of signal peptide and transmembrane domain prediction pipelines. Based on the presence or absence of a signal peptide (predicted by SignalP) and transmembrane domains (predicted by TMHMM), the identified proteins were classified into distinct functional categories. In detail, a cohort of 385 proteins was identified to harbour canonical signal peptides while lacking typical transmembrane segments. These candidates were defined as secretory proteins, which are presumably exported to the extracellular milieu through the conventional secretory cascade. A further 926 proteins lacked identifiable signal peptides but possessed integral transmembrane domains, and were thereby categorised as non-secretory membrane proteins. This protein subset is inferred to participate in fundamental physiological processes, such as substrate transportation, signal perception and cellular structural stabilisation. Additionally, 2897 proteins—lacking both a signal peptide and an intact transmembrane domain—likely constitute soluble non-secretory proteins. In contrast, 132 proteins concurrently carried both signal peptides and transmembrane regions, implying their potential identity as signal peptide-anchored membrane proteins (Figure 9). These findings underscore the value of bioinformatic prediction in dissecting the molecular basis of pathogen–host interactions, while also offering a suite of candidate targets for subsequent functional validation and mechanistic studies of pathogenicity.

#### 2.5.4. Secondary Metabolite Biosynthetic Gene Clusters

Secondary metabolites produced by microorganisms play pivotal roles in environmental adaptation, inter-specific competition and pathogenicity. Nevertheless, the biosynthetic gene repertoire of *P. ananatis*, as an important phytopathogen, has remained systematically unexplored. Herein, whole-genome analysis unveils the metabolic potential of this organism. Specifically, genomic analysis revealed two homoserine lactone gene clusters, encompassing 39 genes. These loci play a central role in quorum sensing and consequently mediate virulence expression. Furthermore, the genome harbors one gene cluster for redox cofactors (e.g., PQQ), spanning 21 genes. This cluster is likely involved in environmental adaptation. Additional secondary metabolite gene clusters were identified, including one thiopeptide cluster (16 genes) linked to interspecific competition, two siderophore clusters (19 genes) essential for host colonization, and one aryl polyene cluster (41 genes) associated with stress tolerance. A summary of all gene clusters is provided in Table 3. Collectively, these findings systematically delineate the secondary metabolic landscape of *P. ananatis*. This work establishes a valuable set of genetic targets and a theoretical foundation for future research.

#### 2.5.5. Pathogen–Host Interaction (PHI)

**Figure 10:**
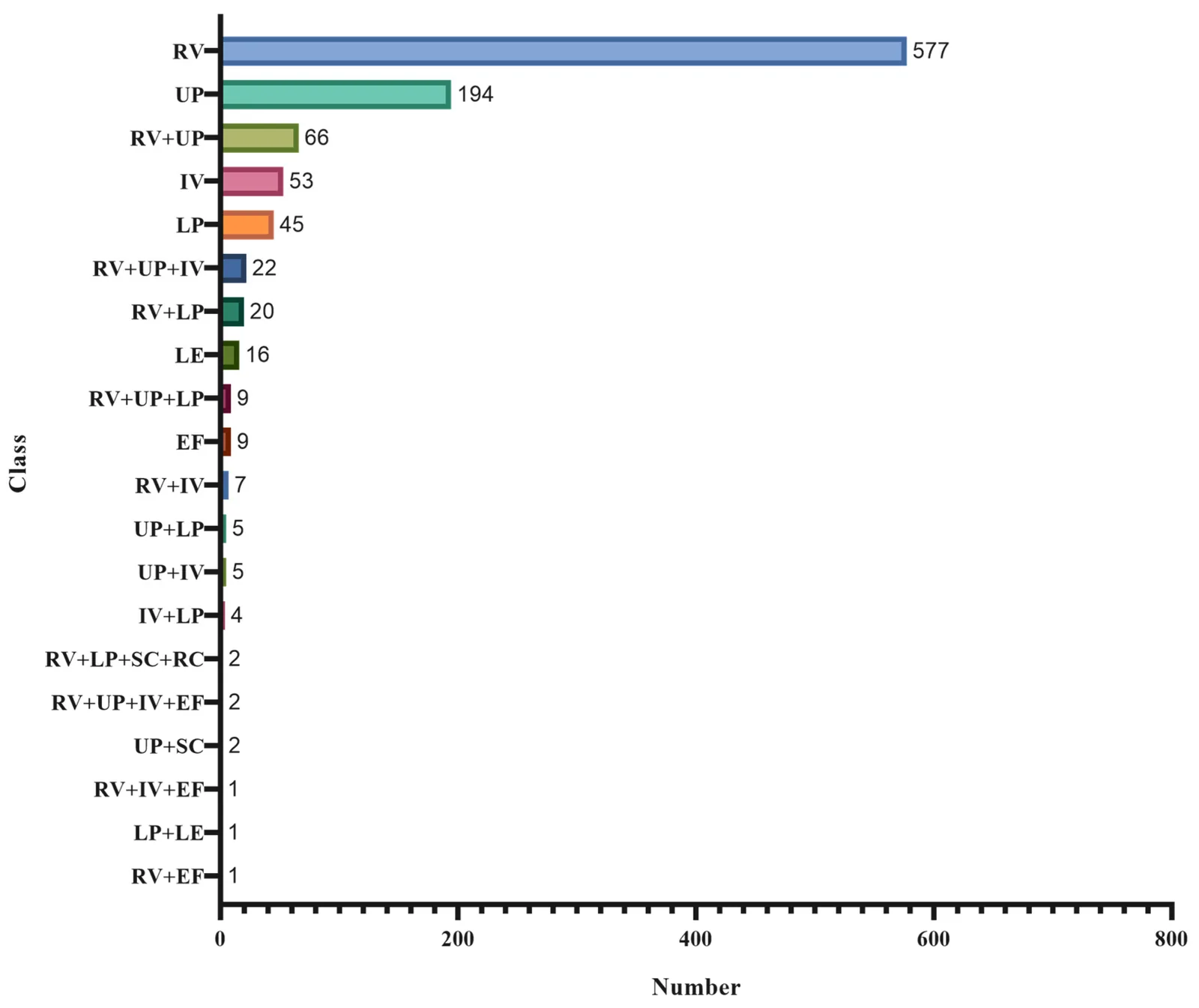
PHI database annotation. RV: reduced virulence, UP: unaffected pathogenicity, IV: increased virulence (hypervirulence), LP: loss of pathogenicity, LE: lethal, EF: effector (plant avirulence determinant), SC: chemistry target: sensitivity to chemical, RC: chemistry target: resistance to chemical.

Although *P. ananatis* is an important phytopathogenic bacterium, the genetic determinants underpinning its virulence remain poorly characterized at the genome-wide scale. In particular, the comprehensive repertoire of virulence factors has yet to be systematically deciphered. The Pathogen–Host Interaction Database (PHI-base) was used to comprehensively annotate and functionally classify its virulence-associated genes via sequence alignment. A total of 1041 homologs of known pathogenicity determinants were identified, revealing an intricate virulence genetic network. Genes causing reduced virulence (RP) upon disruption were dominant (577, 55.42%), indicating that pathogenicity depends on synergistic action of an extensive gene network rather than a few isolated genes. In contrast, canonical effector (EF, RV + EF, RV + IV + EF, RV + UP + IV + EF) genes were extremely rare (13, 1.25%), suggesting *P. ananatis* relies more on cell wall-degrading enzymes, toxins or siderophores for pathogenicity than on type III secretion system-delivered effectors. Additionally, 86 genes associated with complete loss of pathogenicity (LP, RV + LP, UP + LP, IV + LP, LE + LP, RV + LP + UP, SC + RC + RV + LP) and 17 lethal (LE, LE + LP) genes were identified, which are prioritised as potential drug or control targets (Figure 10). This study systematically characterized the virulence-factor atlas of *P. ananatis*, deepening understanding of its pathogenic mechanisms and furnishing a conceptual framework and repository of information for future functional studies and the development of novel control strategies.

#### 2.5.6. Carbohydrate-Active Enzymes

**Figure 11:**
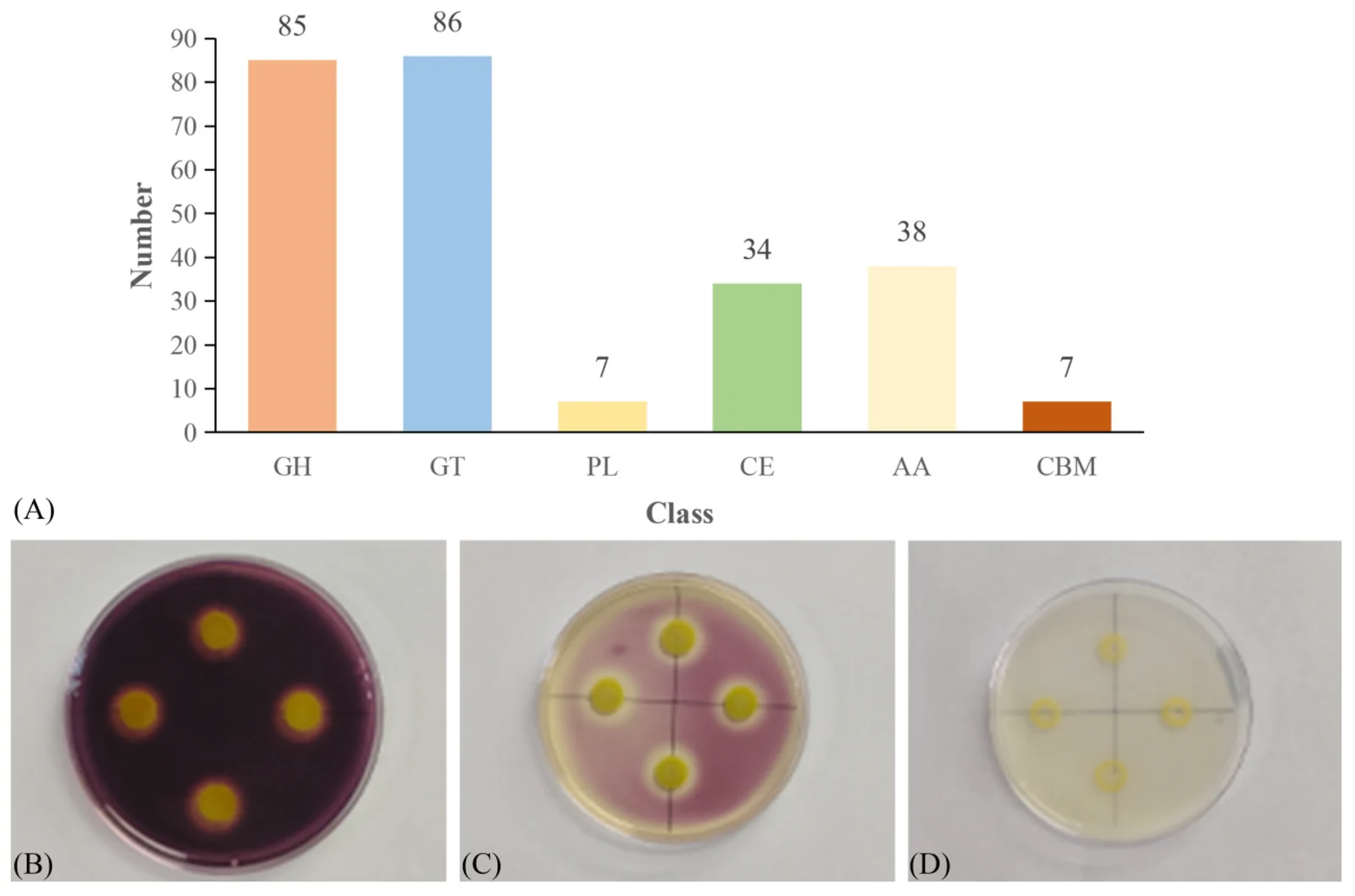
Carbohydrate-active enzymes annotation map and Secretory capability of cell wall-hydrolytic enzymes by the pathogen. (**A**) Carbohydrate-active enzymes annotation map, GH: Glycoside Hydrolases, GT: Glycosyl Transferases, PL: Polysaccharide Lyases, CE: Carbohydrate Esterases, AA: Auxiliary Activities, CBM: Carbohydrate-Binding Modules. (**B**–**D**) Plate assays for cellulase, xylanase and pectate lyase activities produced by the pathogenic isolate, respectively.

Plant cell walls consist of cellulose, hemicellulosic polysaccharides, pectic substances, lignin, and additional structural polymers, and carbohydrate-active enzymes (CAZymes) play pivotal roles in their degradation [38]. In the present study, 257 CAZyme-encoding genes were annotated from the genome of this pathogenic bacterium. These genes were classified into six major families: 86 glycosyltransferases (GTs), 85 glycoside hydrolases (GHs), 38 auxiliary activities (AAs), 34 carbohydrate esterases (CEs), 7 polysaccharide lyases (PLs), and 7 carbohydrate-binding modules (CBMs) (Figure 11A). The strain displays a distinct preference in its CAZyme repertoire. The high abundance and broad subfamily diversity of GHs, including GH1, GH2, GH3, GH5, GH13 and GH43, highlight its strong potential to degrade major polysaccharide backbones. Moreover, the high representation of AAs (notably the AA3 family) and CEs (e.g., CE1, CE7, CE10) suggests a synergistic mechanism for dismantling the complex architecture of the plant cell wall. In contrast, only 7 PLs were detected with limited family diversity. This CAZyme repertoire—enriched in GHs, AAs, and CEs but depleted in PLs—uncovers the cell wall-degrading strategy employed by the pathogen, providing a molecular foundation for further investigations into its pathogenic mechanism.

To preliminarily investigate the pathogenic mechanisms of this isolate, the production of cell wall-degrading enzymes was measured. The results revealed robust xylanase and cellulase activities, indicating a capacity to efficiently degrade hemicellulose and cellulose components of the plant cell wall. Notably, however, no pectate lyase activity was detected. This cellulase/xylanase-rich, pectate lyase-deficient profile may reflect the strategy for cell wall degradation, in which the pathogen bypasses the pectin barrier while directly targeting the structural polysaccharides of the cell wall interior (Figure 11B–D).

## 3. Discussion

With the large-scale cultivation and widespread application of foxtail millet, crop diseases have become increasingly prevalent in agricultural production. This worsening disease pressure is largely attributed to long-term continuous cropping, limited rotation options, and variable resistance among millet cultivars. These diseases have substantially compromised both yield and grain quality.

*P. ananatis* possesses an extraordinarily broad host spectrum and acts as the causative pathogen of multiple economically destructive plant diseases. Representative cases involve diverse host plants, such as fruitlet rot of pineapple (*Ananas comosus*) [39], leaf spot disease of maize (*Zea mays*) [40], leaf spot of sorghum (*Sorghum bicolor*) [41], fruit rot of cantaloupe melon (*Cucumis melo*) [42], stem rot of water bamboo (*Zizania latifolia*) [43], stem necrosis of rice (*Oryza sativa*) [44], inner glume browning of rice [7], and leaf spot disease of rice [45].

In this study, the pathogen responsible for an emerging foliar disease of foxtail millet in Liaoning Province was identified as *P. ananatis*. The isolate was separated from symptomatic leaves and purified, and its virulence was subsequently verified using Koch’s postulates, establishing the isolate as the causal agent. To resolve its taxonomic position, phylogenetic analysis was performed based on the combined sequences of three housekeeping genes (*gyrB*, *infB*, and *rpoB*). Notably, *Pantoea stewartii* subsp. *indologenes* has previously been reported as a causal agent of leaf spot on pearl millet in India [46]. The resulting tree placed the isolate in a clade containing seven reference strains of *P. ananatis* with 100% bootstrap support, clearly separated from *P. stewartii* subsp. *indologenes*. This result firmly assigns FXFM to *P. ananatis*. While molecular markers delineated its phylogenetic identity, a comprehensive biochemical profile of *P. ananatis* has not been previously documented. The bacterium was Gram-negative, oxidase-negative, and arginine dihydrolase-positive; it efficiently utilized maltose, mannitol, and xylose as carbon sources but did not metabolize glucose.

The whole-genome assembly was generated for a pathogenic isolate obtained from diseased foxtail millet. When combined with publicly available genomic data from representative *Pantoea* species, this high-quality genome serves as a valuable genomic resource. It facilitates the exploration of molecular mechanisms underlying pathogenicity and host adaptation across the *Pantoea* genus. Furthermore, these genomic resources support in-depth mechanistic research tailored to foxtail millet cultivation systems. Previous studies have established that the type II secretion system (T2SS) of *Erwinia* and *Xanthomonas* species mediates the extracellular secretion of pectinases and pectate lyases [47,48]. Notably, core genes encoding structural components of the T2SS and type III secretion system (T3SS) are absent from the *P. ananatis* genome [7,26]. Genome annotation revealed that P. ananatis harbors an extensive set of genes encoding cell wall hydrolases. Moreover, annotation against PHI-base identified only 13 effector genes (1.25%). Together, these observations imply that the pathogenic mechanism of *P. ananatis* may not rely on conventional virulence factors secreted via T3SS or T2SS, but instead involves an alternative, yet-to-be-elucidated pathogenic strategy. Beyond secretory systems, clusters of biosynthetic genes linked to virulence play a crucial role in the capacity of *Pantoea* species to infect diverse hosts. Through genome-wide profiling of secondary metabolite biosynthetic gene clusters, this study systematically identified multiple key biosynthetic loci in this *P. ananatis* isolate, including those for homoserine lactones, the PQQ redox cofactor, thiopeptides, siderophores, and aryl polyenes. These results further establish their critical roles in environmental adaptation, interspecific competition, host colonization, and the regulation of bacterial virulence.

To establish colonization and disseminate within the host, *P. ananatis* relies on the initial secretion of an array of cell wall-degrading enzymes (CWDEs). These CWDEs hydrolyze the structural components of the host cell wall, facilitating subsequent invasion of host tissues. The repertoire of CWDEs deployed by *P. ananatis* remains incompletely characterized. In this study, the bacterium exhibited robust xylanase and cellulase activities, indicating its capacity to efficiently degrade hemicellulose and cellulose components of the plant cell wall. Notably, however, no pectate lyase activity was detected, a finding consistent with the genomic observation that this strain harbors only seven polysaccharide lyase (PL)-encoding genes with limited diversity. This paucity of pectin-degrading enzymes aligns with the absence of detectable pectinolytic activity in vitro, and may reflect the cell wall degradation strategy employed by this pathogen. In foxtail millet, the cell wall is rich in arabinoxylan and cellulose, with pectic polymers concentrated in the middle lamella and primary wall. The CAZyme profile of this isolate is rich in cellulase and xylanase and deficient in pectate lyase. This profile may favor degradation of xylan-rich and cellulose-rich vascular and sclerenchyma tissues, thereby aiding entry into xylem and phloem. It may also limit the release of pectic oligogalacturonides, which can act as damage-associated molecular patterns and trigger immune signaling [49,50,51]. Such a profile may also reduce early activation of pattern-triggered immunity during systemic movement, although this hypothesis requires functional validation.

Colonization and distribution assays of the pathogen in foxtail millet plants demonstrated that *P. ananatis* can migrate upward from the initial infection site in the roots and establish systemic colonization in the leaf sheaths and blades. These findings confirm that the emerging foxtail millet disease caused by *P. ananatis* represents a typical systemic infection. A preliminary host-range assay further demonstrated that the *P. ananatis* strain characterized in this study is capable of infecting not only foxtail millet, but also peanut, maize, rice, and *Arabidopsis thaliana* (Figure S1).

The systemic colonization of roots, leaf sheaths, and leaf blades observed in this study provides preliminary guidance for field management. If root infection is a primary entry route, avoiding waterlogging, reducing root wounds, and regulating soil moisture may restrict pathogen ingress. If leaf sheaths and blades have been colonized systemically, early monitoring and removal of diseased plants before symptom expansion can reduce secondary spread. Crop rotation should be tailored to host range and residue survival. Chemical control should target root infection and early systemic colonization rather than late foliar symptoms. Resistance breeding can prioritize genotypes that restrict systemic colonization or possess stronger cell wall-based defenses [52,53,54].

From a plant protection perspective, the actionable strategies for this disease should integrate exclusion, seed health, cultural practices, and targeted chemical or biological control. Because systemic infection can originate from roots and spread through vascular tissues, management should prioritize pathogen-free seed and transplants, sanitation, and removal of infected residues. Crop rotation with non-host cereals or dicot crops may reduce soil and residue inoculum, although the broad host range of *P. ananatis* limits rotation options [7,52]. Chemical optimization should select systemic fungicides, with resistance inducers used as supplements. Moreover, seed treatment, soil drenching, and foliar applications should be timed to early infection stages, with rotation of active ingredients. Biological control agents and microbial antagonists may further suppress epiphytic and soilborne inoculum [52,53]. Resistance breeding should target vascular colonization, cell wall reinforcement, and immune recognition of conserved bacterial elicitors, using the genomic and phenotypic data reported here to screen millet germplasm for reduced systemic spread [54].

Collectively, these findings offer critical insights into the infection route, tissue adaptability of *P. ananatis*, as well as the interplay between pathogen and host. These findings also establish a theoretical foundation for functional characterization of virulence genes, identification of candidate control targets, and formulation of environmentally friendly strategies aimed at limiting systemic colonization by *P. ananatis* in foxtail millet.

## 4. Materials and Methods

### 4.1. Isolation and Purification of the Pathogen

Diseased leaf specimens were collected in 2022 from the foxtail millet cultivar ‘Jinmiao K1’ grown in Fuxin City, Liaoning Province, China. The pathogen was isolated using conventional tissue-isolation methods [55]. Leaves exhibiting disease symptoms were collected from the field and rinsed using sterile dH_2_O. Small segments were excised from the margins of lesions and surface-disinfected with 75% ethanol for 1 min. After rinsing three times with sterile dH_2_O, the leaf segments were transferred into sterile 1.5 mL microcentrifuge tubes. Subsequently, 1 mL of sterile distilled water was incorporated, and the mixture was allowed to stand for 2–3 min. After a 10,000-fold dilution, the supernatant was spread onto LB agar plates (5 g yeast powder, 10 g peptone, 10 g sodium chloride, and 15 g agar per liter). Incubation was conducted at 25 °C for 48 h, after which well-separated colonies were randomly selected and restreaked onto fresh agar medium to yield clonal isolates. Isolated single colonies were inoculated into liquid medium and cultured to prepare a bacterial suspension. Glycerol was then added to a final concentration of 20%, and the strain stock was cryopreserved at −80 °C.

### 4.2. Pathogen Identification

To identify the pathogen in accordance with Koch’s postulates, the isolate was tagged with GFP. The GFP-tagged strain was inoculated onto foxtail millet cultivar ‘Jinmiao K1’ seedlings by soil infiltration inoculation. In brief, each seedling received 500 μL of bacterial suspension (OD600 = 1.0) in the root-zone soil. At least ten seedlings were inoculated per treatment, with three independent biological replicates. Mock inoculation with sterile dH2O served as the negative control. After inoculation, plants were maintained in a greenhouse at 25 °C. Thirteen days post-inoculation (dpi), symptoms were photographed. The pathogen was then re-isolated from symptomatic leaves and its taxonomic identity was verified, thereby fulfilling Koch’s postulates [56].

### 4.3. Multilocus Sequence Analysis (MLSA) for Pathogen Identification

The bacterial strains were cultured until reaching the exponential phase (OD_600_ = 0.5), after which cells were harvested for genomic DNA extraction. Total DNA was extracted with the BioFlux bacterial gDNA extraction kit (Coolaber, Beijing, China). Three housekeeping genes (*gyrB*, *infB* and *rpoB*) were amplified with specifically designed primers listed in Table 4, as previously described [14,43,57]. The resulting PCR products were size-fractionated on a 1% (*w*/*v*) agarose gel, purified with a commercial DNA purification kit (Coolaber, Beijing, China), and subjected to Sanger sequencing. The obtained sequences were used for phylogenetic analysis and species-level identification within the genus *Pantoea*.

### 4.4. Biochemical Characterization of the Pathogen

The isolate stored at −80 °C was streaked onto fresh LB agar plates and incubated at 28 °C for 24–48 h to obtain individual colonies, which were then used for subsequent experiments. Gram staining was carried out using the protocol of Bartholomew and Mittwer [58]. For oxidase activity assessment, an oxidase test strip (made by Qingdao Hope Bio-Technology Co., Ltd., Qingdao, China) was moistened with sterile distilled water, and a single colony of fresh pure culture was evenly smeared onto it. Development of a blue or blue-purple color within 30 s was interpreted as a strong positive result; no color change within 2 min indicated a negative result. Biochemical characteristics were determined using the direct inoculation method. A single colony of fresh pure culture was aseptically inoculated into a series of micro-biochemical identification tubes (made by Hangzhou Microbiological Reagent Co., Ltd., Hangzhou, China), including tests for carbohydrate fermentation, gelatin liquefaction, hydrogen sulfide production, nitrate reduction, DNase activity, acetamide utilization, arginine dihydrolase activity, and Simmons citrate utilization. Following inoculation, tubes were wrapped with Parafilm and held at 28 °C for 24–48 h. Color changes were monitored, and results were recorded and interpreted according to the manufacturer’s standards.

### 4.5. Phylogenetic Tree Construction

To elucidate the evolutionary placement of the strain, a ML tree was inferred from the concatenated sequences of the three housekeeping genes (*gyrB*, *infB* and *rpoB*) derived from various *Pantoea* species and subspecies. Sequence alignment, curation, trimming, and concatenation were performed using the PhyloSuite platform [59] integrated with MAFFT, MACSE, Gblocks, and the Concatenate Sequences function. The final ML analysis was conducted with IQ-TREE v1.6.12 [60], employing 1000 bootstrap replicates to assess nodal support. *Tatumella morbirosei* was designated as the outgroup to root the phylogeny.

### 4.6. Visualization of the Pathogen’s Infection Pathway in Foxtail Millet Seedlings

To track the infection pathway, a GFP-tagged derivative of the wild-type *P. ananatis* strain was constructed. The broad-host-range plasmid pDSK, harboring the *gfp* gene, was introduced into bacterial cells via electroporation. Competent cells were mixed with the plasmid, transferred to a pre-chilled electroporation cuvette, and subjected to a single pulse of 2.5 kV for 5 ms. Following electroporation, cells were recovered in antibiotic-free LB medium for 2 h and subsequently plated onto selective LB agar containing the appropriate antibiotic to isolate transformants. Clones exhibiting successful GFP expression, as verified by fluorescence microscopy, were selected for further experimentation. Bacterial cells were cultivated to logarithmic phase, harvested, and resuspended to OD_600_ = 1.0. One-month-old foxtail millet seedlings were inoculated via root immersion in the bacterial suspension. At 13 dpi, the distribution of GFP-tagged bacteria within the seedlings was visualized under 365 nm excitation light to elucidate the colonization pattern and infection pathway.

### 4.7. Whole-Genome Sequencing and Gene Annotation

Whole-genome sequencing and database construction were conducted by Shanghai OE Biotech Corporation (Shanghai, China) using Oxford Nanopore sequencing technology. Raw Nanopore reads were processed for de novo genome assembly using Unicycler v0.4.9 [61]. Bacterial protein-coding genes were predicted with Prodigal v2.6.2 (https://github.com/hyattpd/prodigal/wiki, accessed on 26 September 2026), while tRNAs and rRNAs were identified using tRNAscan-SE 2.0 [62] and Barrnap v0.9 (https://github.com/tseemann/barrnap, accessed on 26 September 2026), respectively. Secondary metabolic biosynthetic gene clusters were annotated via the antiSMASH pipeline [63]. For functional gene annotation, all predicted coding sequences were aligned against the GO, eggNOG/COG, KOG and PHI-base databases using BLAST (https://ftp.ncbi.nlm.nih.gov/blast/db/, accessed on 26 September 2026). Circular genome visualization was generated with the circlize package in R [64]. Plasmids were annotated using Prokka (https://2017-cicese-metagenomics.readthedocs.io/en/latest/prokka_tutorial.html, accessed on 26 September 2026) [65].

### 4.8. Assay of Cell Wall-Degrading Enzyme Activities

Cell wall hydrolase activities were determined following a previously described protocol [66]. In brief, extracellular pectate lyase, cellulase, and xylanase activities were examined on three selective agar media: pectate lyase detection medium (5 g·L^−1^ citrus pectin, 5 g·L^−1^ tryptone, 0.2 g·L^−1^ MgSO_4_, 1 g·L^−1^ K_2_HPO_4_, and 17 g·L^−1^ agar); cellulase detection medium (5·g L^−1^ microcrystalline cellulose, 10 g·L^−1^ tryptone, 5 g·L^−1^ yeast extract, 10 g·L^−1^ NaCl, and 17 g·L^−1^ agar); and xylanase detection medium (5 g·L^−1^ xylan, 10 g·L^−1^ tryptone, 5 g·L^−1^ yeast extract, 10 g·L^−1^ NaCl, and 17 g·L^−1^ agar). A 10 μL bacterial inoculum (adjusted to 10^9^ CFU·mL^−1^) was spot-inoculated onto each medium plate. Pectate lyase plates were maintained at 25 °C for 48 h, flooded with 10 mL of 1% (*w*/*v*) cetyltrimethylammonium bromide (CTAB) solution, and scored for hydrolysis halos 10 min post-treatment. Cellulase and xylanase plates were incubated under identical conditions, stained with 10 mL of diluted iodine, and examined for clear zones after 2 min.

## 5. Conclusions

This is first record in China of foxtail millet leaf disease caused by *P. ananatis*. Fluorescence-based tracking confirmed that the bacterium migrates and disseminates within the root system, leaf sheaths, and leaves. These observations establish that the disease is systemic. Moreover, the genome of the isolated pathogen was sequenced using Oxford Nanopore technology, yielding a high-quality, fully assembled and annotated assembly. All genomic data generated in this study were deposited in NCBI public databases. BioProject PRJNA1496023, BioSample SAMN61665871, assembled genome GenBank entry JCBCVY000000000, and raw sequencing SRA data SRR39728977. Next its cell-wall-degrading enzyme activities were profiled. The strain efficiently hydrolysed hemicellulose and cellulose components of the plant cell wall, whereas pectate lyase activity was undetectable. This finding aligns with the CAZymes database annotation. This cellulase/xylanase-rich, pectate lyase-deficient profile may reflect the strategy for cell wall degradation, in which the pathogen bypasses the pectin barrier while directly targeting the structural polysaccharides of the cell wall interior. These results are poised to inform programs for resistance improvement and guide the targeted molecular optimization of agrochemicals.

## Figures and Tables

**Table 1:** Biochemical characteristics of pathogenic bacteria.

Biochemical Characteristic	Result
Oxidase	−
Glucose oxidation	NRO^−^
Maltose	+
Mannitol	+
Xylose	+
Gelatin hydrolysis	−
H_2_S production	−
Nitrate reduction	−
DNase	−
Acetamide	−
Arginine dihydrolase	+
Simmons’ citrate	+

Note: +, positive reaction; −, negative reaction; The glucose utilization was scored as F (fermentative), O (oxidative), or NR0^−^ (not utilized; no color change in either tube).

**Table 2:** Plasmid Statistics Table.

Category	Length (bp)	GC Content (%)	Sequencing Depth
Property	304,093	52	0.72

**Table 3:** Prediction of genomic metabolic gene clusters.

Region	Type	From	To	Gene Number
Region 1	Homoserine lactone	2,478,675	2,497,839	18
Region 2	Redox-cofactors	2,602,533	2,624,698	21
Region 3	Homoserine lactone	3,091,192	3,111,803	21
Region 4	Thiopeptide	3,171,291	3,197,556	16
Region 5	Siderophore	3,991,084	4,002,441	8
Region 6	Aryl polyene	4,054,526	4,098,122	41
Region 7	Siderophore	4,392,866	4,407,271	11

**Table 4:** The primers used in this study.

Gene	Primer	Primer Sequence	Product Size (bp)	Reference
*gyrB*	pagyrB-F	5′-TACGGGAGATACCGATGC-3′	673	[43]
	pagyrB-R	5′-GCGTGCGGCGTCAATAAT-3′		
*infB*	infB 01-F	5′-ATCATGGGCCACGTTGACCA-3′	1124	[57]
	infB 02-R	5′-ACGGAGTAGTAACGCAGATCCA-3′		
*rpoB*	rpoB-F	5′-GGCTGAAAACCAGTTCCGCGT-3′	944	[14]
	rpoB-R	5′-CCAGAGAGACACACGGCATCT-3′		

## Data Availability

The data have been deposited in the laboratory of Y.-H.Z. and are accessible on request. All genome datasets produced from this work have been uploaded to NCBI’s open-access databases. Detailed access codes are provided as follows: BioProject PRJNA1496023, BioSample SAMN61665871, assembled genome GenBank entry JCBCVY000000000 and raw sequencing SRA data SRR39728977. All these data resources will be fully accessible to the public after the official online release of this manuscript.

## Supplementary Materials

The following supporting information can be downloaded at: https://www.mdpi.com/article/10.3390/plants15192986/s1, Figure S1: Host range determination.
