# Supplementary material for: A New Leaf Bacterial Disease of Foxtail Millet Caused by Pantoea ananatis in China
Source: Plants (Basel). 2026 Sep 30;15(19):2986. doi: 10.3390/plants15192986 (PMC13666582; doi:10.3390/plants15192986)
Supplement: Supplementary file 1 [file plants-15-02986-s001.zip › Figure S1.pdf]

Materials and Methods

The plant materials comprised peanut cv. Fuhua 30, maize cv. Fuyu 1315, rice cv. Longjing 31, and wild-type *Arabidopsis thaliana*. A GFP-labeled strain FXFM was introduced into seedlings of each species through soil drench inoculation. Each seedling received 500 μL of bacterial suspension at OD_{600} = 1.0 in the root-zone soil, and three independent biological replicates were established per treatment. Following inoculation, plants were kept in a greenhouse at 25°C. At 13 days post-inoculation (dpi), symptoms were photographed and documented.

Results

As an initial step to define the host range of *Pantoea ananatis*, GFP-labeled bacterial cells were delivered by soil drench inoculation to seedlings of peanut, maize, rice, and *Arabidopsis thaliana*. At 13 days post-inoculation, fluorescence was examined. The pathogen successfully infected all four species (Figure S1).

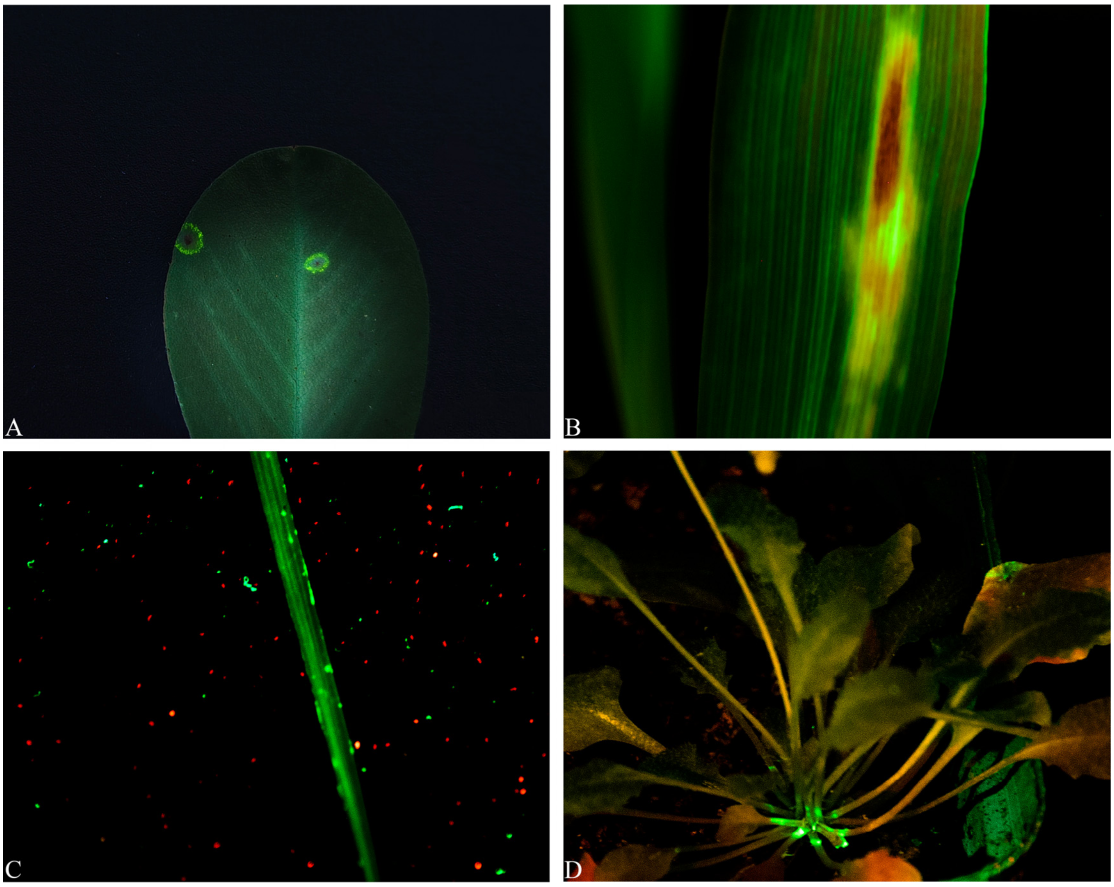

Figure S1 Host range determination

Note: A Peanut， B Maize， C Rice， D *Arabidopsis thaliana*
